# Exposure Science: A View of the Past and Milestones for the Future

**DOI:** 10.1289/ehp.0901634

**Published:** 2010-03-22

**Authors:** Paul J. Lioy

**Affiliations:** Environmental and Occupational Health Sciences Institute, University of Medicine and Dentistry of New Jersey–Robert Wood Johnson Medical School, Piscataway, New Jersey, USA

**Keywords:** chemical and biological agents, chromium, epidemiology, exposome, exposure assessment, exposure science, risk management, source-to-dose modeling

## Abstract

**Background:**

The study of human exposure to environmental toxicants has evolved as a scientific field over the past 30 years.

**Objectives:**

This review provides a historical perspective on the growth of exposure science as a field, with some emphasis on the results from initial observational studies in obtaining information needed for generating hypotheses on significant human contact with environmental agents, testing the performance of models, and reducing exposures to protect public health.

**Discussion:**

Advances in activity pattern and behavioral research that established a suite of variables needed to accurately define contact and factors that influence contact are also discussed. The identification and characterization of these factors have played a pivotal role in the growth of the field and in developing exposure reduction strategies. Answers to two key questions on the relevance and fundamental value of exposure science to the fields of environmental health and risk management are presented as a path forward: *a*) What does one do with such exposure information? *b*) What roles does exposure science play in situations beyond observational analyses and interpretation?

**Conclusions:**

The discussion identifies the need for more focused use of observational studies of exposure for epidemiologic analyses. Further, the introduction and use of new tools and approaches for hypothesis testing that can improve the use of exposure science in prevention research for risk management is needed to affect the source-to-effect continuum. A major restructuring of the field is not required to achieve innovation. However, additional resources for training and education are required to ensure that the potential for exposure science to play a central role in reducing and preventing excess risk within environmental/occupational health is achieved.

As we begin the second decade of the 21st century, environmental science and environmental health science are well-defined fields of research. For instance, environmental science includes understanding the sources of toxicants and the processes that release and transport them though air, water, soil, or food and has applications to sustainability. Environmental health science describes the processes and effects that occur after the human body has received a toxicant, including mechanistic research in toxicology as well as epidemiology or clinical practice. However, neither environmental science nor environmental health science directly addresses the fundamental issues of whether and how human contact with toxicants occurs after release into the environment or workplace.

The relatively new field of exposure science provides information and tools to bridge or to directly link the above disciplines by quantifying and characterizing the conditions for contact with toxicants. For example, after a chemical, physical, or biological toxicant enters an environmental medium (e.g., by atmospheric dispersion), if a person’s behavior or activities puts him or her in contact with the toxicant, the human body can be exposed through inhalation, ingestion, or dermal route. Subsequently, the material can be absorbed or adsorbed, resulting in a dose and potentially a disease outcome. The importance of exposure science in defining those links and protecting public health, however, is still not universally understood. Clearly, throughout the second half of the 20th century, workers began to be protected by wearing personal protective equipment (PPE) or adding near-field engineering or administrative controls on the source. In contrast, PPE is not a norm in environmental settings. Thus, exposure science is essential for eliminating or reducing contacts with toxicants or for altering people’s activities/habits before a problem arises. The applications within environmental settings are more complex than for occupational settings, including the fact that toxicant concentrations are usually much lower. In this review I provide information on the status of the field and my insights on what needs to be accomplished to make the field more visible and make the research results more usable by a wide range of scientific disciplines and policy makers.

In 1987 the first National Research Council (NRC) Committee on Exposure met to discuss inhalation exposure to air pollution. It was partly an outgrowth of research conducted in the Harvard Six Cities study and in indoor air pollution issues, for example, environmental tobacco smoke and radon (Dockery et al. 1993; NRC 1981). The recommendations of the multidisciplinary NRC committee set forth new research directions, and the evaluation also helped define the core principles for the entire field of exposure. The activities of the committee accelerated the birth of the International Society of Exposure Science as well as a scientific journal focused on exposure (NRC 1991). At the time, exposure assessment had been considered only a practical analysis tool invented primarily to support the field of risk assessment (NRC 1983). However, the actual roots of the field could be found in industrial and occupational hygiene (Gochfeld 2005; Harris 2000). Further, the general concept of contact with a toxic agent can be traced back even deeper into the history of occupational medicine, with the qualitative aspects of exposure being traceable to the treatise *De Morbis Articum* written by Ramazzini in 1688 (Lioy 2007; Ramazzini 1964). The main concept derived from occupational/industrial hygiene was the need to focus on personal contact with the toxicant of concern.

The work of the committee solidified a basic equation of exposure:


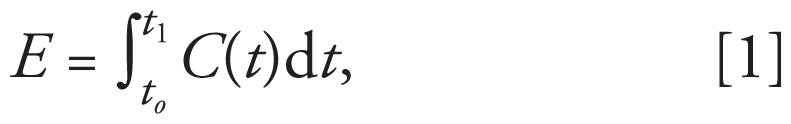


which showed that exposure (*E*) is a function of both concentration (*C*) and intervals of time (d*t*), but the form can also be a linear summation of discrete exposures (NRC 1991). However, the most important point is that duration and frequency of exposure must also be coupled to a biologically relevant time of contact for any disease outcome before *E* can have any meaning in terms of defining human exposure–response relationships. Thus, exposure science must consider toxicologic and epidemiologic information to focus hypotheses. This is understandable, because the types of contacts that are important for determining the potential for long-term effects will not necessarily be the same as those for acute effects.

During the NRC discussions, I published an article on the concepts of source to dose, showing the central role human exposure has in linking traditional environmental science with the fields of toxicology, epidemiology, and risk assessment (Lioy 1990). The article included a process continuum from source to human health that focused on the location of the field within the environment and health processes. It did not provide details on each field to the left and the right of exposure science (the most recent revision of the continuum is shown in Figure 1), but identifies major processes and points to issues of risk management and prevention.

In 1990, Wayne Ott published a paper on exposure assessment research at the U.S. Environmental Protection Agency (EPA) (Ott 1990). An update (Lioy 1999) built on the work of others, including the ideas presented in the article on the “birth of a new science” (Ott 1995). These and other events were summarized recently in my editorial “Time for a Change: From Exposure Assessment to Exposure Science” (Lioy 2008).

The definition of exposure science was published in the *Journal of Exposure Science and Environmental Epidemiology* by Dana Barr (2006):

It studies human contact with chemical, physical or biological agents occurring in their environments, and advances knowledge of the mechanisms and dynamics of events either causing or preventing adverse health outcomes.

Today the field is at a crucial juncture in its development, as the need for exposure-related research is expanding throughout the world (Barr 2006; Lioy et al. 2005; Sheldon and Cohen-Hubel 2009; Weis et al. 2005; Wilson and Suk 2005). However, the resources and funding required to support the science are still inadequate when compared with resources provided for the other components of environmental health sciences. For example, in the United States, the National Institute of Environmental Health Sciences (NIEHS, personal communication) is funded at a level of > $680 million year, but its Exposure Biology Program, a special initiative on a subgroup of exposure research needs, totals only about $30 million. Similarly, the National Exposure Research Laboratory (NERL) of the U.S. EPA is funded only at a level of about $50 million/year (EPA-NERL, personal communication). These numbers indicate significant underfunding of the field and probably reflect the situation throughout the world. Further, to move the field forward during the first half of the 21st century, both the number of exposure science professionals and their skill sets must expand beyond what was sufficient for the past 25 years. However, in contrast to toxicology, there is no federally funded training program dedicated primarily to this component of environmental health science, despite its potential to serve as a transdisciplinary bridge between hazardous wastes, nanotechnology, consumer products, sustainability, alternate fuels and energy, and other critical research areas.

The lack of a steady stream of investigators coming into the field is also a problem for proposal submissions and review. Basically, there are not enough peers on grant and contract review panels to provide a balanced evaluation of exposure science proposals, which are often inappropriately reviewed as if they were environmental science or toxicologic studies. This results in a lack of understanding of exposure science research and a hesitancy to fund projects that advance the field. Without adequate, dedicated training programs and funding mechanisms, it will be difficult to increase the recognition of the utility of exposure science; consequently, future risk assessments and other applications will still have major gaps in exposure information. Further, if the concept of the “exposome”—“all environmental exposures from conception onwards (including exposures from diet lifestyle, and endogenous sources) as a quantity of critical interest to disease etiology”—continues to evolve, investigators in the field will need more skill sets to ensure that there are tools and approaches available to integrate and interpret different types of data (Wild 2005).

## Perspective on Research

With the above in mind, current and future scientific research issues for the field include the identification and evaluation of populations in contact with toxicants in countries around the world and development of new technologies and biomarkers to more accurately characterize exposures and solve problems by reducing or preventing exposure. For each, the needs include better measurement and analytical equipment, human behavior and activity pattern analyses, automated sensors, robotics, field simulations of exposure, and source-to-exposure and dose-modeling systems. The last three tools are important because of the increasing levels of scrutiny being applied to all human studies before approval is granted, including exposure studies, by institutional review boards associated with agencies and universities. Specific examples include *a*) measuring or simulating exposures that can occur among toddlers, pregnant women, or the elderly, because it is difficult to conduct multiroute personal monitoring on such subjects (Lebowitz et al. 2008; Shalat et al. 2007; U.S. EPA 2008c); and *b*) augmenting the tools being developed in chemical toxicity testing with a parallel set of tools that can be used to detect low levels in the community and prioritize exposures for subsequent application in risk assessment (Cohen Hubal et al. 2010; Sheldon and Cohen-Hubel 2009). These are areas where innovation can improve our ability to address critical environmental health issues.

### Observational studies of human exposure

Many types of studies of human exposure have included laboratory analyses and computer simulation modeling. However, the growth of the field truly began with the design and completion of field studies. In the 1980s many of the important hypotheses and applications were associated with observational studies (Wallace et al. 1986). This was not surprising, because the collection of personal monitoring and biological monitoring samples provided basic information on contact with toxic materials. Observational studies were warranted at the time, with many of the initial projects associated with reducing exposure to indoor air pollution (NRC 1981). However, there was also a significant engineering influence on the field because of the need for ventilation to at least reduce the levels of air pollutants indoors. The definition of observational studies for the purposes of characterizing human exposure is the collection of personal environmental samples, data, and information from participating volunteers in their everyday environments as they go about their normal activities.

A key part of this definition is that investigators do not purposely expose a subject or volunteers to anything not present or used in their everyday environment. The investigators are passively involved in the lives of the subjects from which they collect samples and data on the chemical, physical, and biological world around them. Programs such as the Total Exposure Assessment Methodology (TEAM) studies (many types and locations) and The National Human Exposure Assessment Survey (NHEXAS) were designed and completed in the United States to acquire such information on how people come in contact with toxicants (Clayton et al. 1993; Pellizzari et al. 1995; Sexton et al. 1995; Wallace et al. 1986). These two studies, as well as other exposure studies conducted around the world, helped establish a baseline on multiroute and multipathway exposures to environmental contaminants (Cohen-Hubal et al. 2000; Hoffmann et al. 2000a, 2000b; Jantunen et al. 1998; Korrick et al. 2000; Weisel et al. 2005; Williams et al. 2000a, 2000b). They provided data needed to reduce uncertainties in the characterizations of exposure and identified ways to reduce exposure—for example, dust control, home repairs, product replacement, and revised regulatory strategies. For those in epidemiology and medicine, such studies represented the types of exposure science investigations that are needed to test hypotheses. Subsequently, the information can be used to improve population-and individual-based human exposure–health response investigations.

The U.S. Food Quality Protection Act (FQPA 1996), passed in 1996, provided new reasons for conducting observational exposure studies (U.S. EPA 2008a). These studies are designed within a framework for evaluating aggregate exposure or cumulative exposures. The specific focus of the FQPA was pesticides, but the regulations can be used to evaluate many other toxicants. As defined, aggregate exposure is the extent of contact and exposure of a defined population to a given chemical by all relevant routes and all relevant sources, and cumulative exposure is the extent of contact or exposure of a defined population to multiple chemicals with common toxic effects found in at least one media and one or more sources (U.S. EPA 2001, 2003). However, specific exposure routes have often been prematurely discounted, which can lead to errors. Thus, both mechanistically based observational studies as well as controlled exposure studies need to ensure that the routes of exposure are selected wisely to characterize population-based aggregate and cumulative exposure and human exposure–response studies.

### Human behavior and activities

Research on human behavior or activities is an essential component of modern and future exposure science. Both mechanistic and field studies have been necessary to reduce uncertainties in levels of exposure for a compound or class of compounds. Included are contacts that can lead to inhalation exposures to resuspendable materials, for example, athletic activities on turf (grass or artificial).

Current observational studies of personal contact with pollutants are being augmented by tools that truly set exposure science apart from environmental science or environmental quality, namely, the mechanistic studies of human activity patterns and behavior in adults and children (Freeman et al. 1997; Klepeis et al. 2001; Reed et al. 1999; Schwab et al. 1991; Zartarian et al. 1997).

Historically, questionnaires have been employed in both occupational and environmental epidemiologic studies and studies of human behavior (Lebowitz et al. 1989). Questionnaires will always have a place in observational exposure research and are augmented by data sets with anecdotal observations and case reports of significant exposures. Information on activity and behavior from these sources were qualitative or semiquantitative in nature. Then investigators developed a new set of tools based on daily diaries and then videotaping of subjects. The results were digitized for quantitative analysis of activities and behaviors associated with continuous or periodic exposure (Freeman et al. 1997, 2001a, 2001b; Ko et al. 2007; Rodes et al. 2001; Royster et al. 2002; Schwab et al. 1991; Zartarian et al. 1997). Each tool has been used to improve observational exposure studies and information in the U.S. EPA’s *Exposure Factors Handbook* (under revision), which is used as a resource by risk assessors or managers (Moya and Phillips 2002; U.S. EPA 1997). Shalat et al. (2007) have employed these data to develop the Pre-toddler Inhalable Particulate Environmental Robotic sampler (PIPER). It mimics the activities of toddlers and young children in locations where actual exposure to toxicants may occur and avoids the use of actual personal monitoring of a toddler in an exposure situation, for example, to residential pesticides. This is an important advance, because the use of toddlers in personal monitoring studies has been questioned and has led to an ethics controversy summarized by Barrett (2009). It also led to the publication of new ethics guidelines for observational exposure studies in general by the U.S. EPA (2008c). PIPER and similar innovative tools could advance the field by minimizing concerns about conducting personal exposure studies on toddlers and could be an effective tool for inhalation and dermal exposure analyses in epidemiologic studies.

A national database, the Consolidated Human Activity Database (CHAD), is available for large population-exposure model simulations (McCurdy et al. 2000). This semi-quantitative information is used to improve the accuracy of exposure characterizations and identify individuals and populations at highest risk for exposure. However, these data are not situation specific and may not apply to sentinel populations, for example, pregnant women.

Global positioning system (GPS) is a tool that can help track subjects in observational exposure studies (Elgethun et al. 2007). These data can be used as inputs to exposure models to understand the significance of human activities and behaviors on contact and estimate distributions of exposure. GPS data are coupled with personal monitoring, microenvironmental monitoring, and activity-pattern data (Duan 1991; Georgopoulos et al. 2006; Glen et al. 2008; Isakov et al. 2007; McKone 1991; Price and Chaisson 2005; Roy et al. 2003; U.S. EPA 1992; van Wendel de Joode et al. 2005).

### Biomonitoring

Biomonitoring has become a component of observational exposure characterization in the United States and other countries (Barr et al. 2005; Henderson et al. 1992; Hoffmann et al. 2000a; Maier et al. 2004). At the same time, biological exposure indices (BEIs) are used routinely to augment the threshold limit value guidelines to identify a worker exposed to toxicants by analyzing biological specimens [American Conference of Governmental Industrial Hygienists (ACGIH) 2009].

Biomonitoring is used by the Centers for Disease Control and Prevention (CDC) to provide a baseline of body burden within the general population for > 100 organic substances and for trace elements (CDC 2009; Kieszak et al. 2002). The distributions of these agents are listed periodically and provide information on the prevalence of various toxicants released into the air, water, soil, and food that enter the body by one or more routes of exposure. Biomonitoring information is an integral part or sum of the contributions from one or more routes of exposure. Clearly, the dose measured depends on the biological sampling media. For example, breath analysis is used to examine the levels of a toxicant within a short period of time after exposure, which would include short-lived or quickly metabolized toxicants. In contrast, measurements of toxicants in the urine or blood provide information on the body burden that occurred for compounds that can pass though the body relatively quickly or compounds that are stored or metabolized over a much longer period of time (Henderson et al. 1992). Biomonitoring data simply indicate that a person has been exposed, unless other data are provided to help determine the routes and pathways of exposure. The success of the exposome concept will be contingent partially on the successful linkage of these components of exposure science (Wild 2005).

Source-to-dose models will help unravel external exposure and internal dose in the future as more pharmacokinetics information is used to develop inverse-modeling tools (Georgopoulos et al. 2009; Mosquin et al. 2009). The goal is to reconstruct the intensity and routes of exposure for the toxicant of concern and ultimately the source. Analyses can be completed for various systems and biological markers using a carefully selected suite of variables from those shown in Figure 2. A significant scientific breakthrough will occur when the appropriate variables are measured simultaneously with biomarkers, and the results are routinely used to reconstruct the route of exposure with reasonable levels of certainty (Georgopoulos et al. 2009; Mosquin et al. 2009; Roy and Georgopoulos 1998). Such data sets and associated analyses will be essential for streamlining risk-reduction strategies.

From the preceding, one can identify at least six possible purposes of observational data, including

- Identifying sources (e.g., lead from battery-powered vehicles, mosquito misters)
- Selecting sentinel populations (e.g., pregnant women)
- Identifying the highest-exposed individuals
- Defining the activities and behaviors that yield exposures
- Defining the magnitude and frequency of exposure
- Identifying exposure–response relationships.

These types of data are necessary to improve the results of epidemiologic studies and uncertainties in risk assessments.

**Figure 1:**
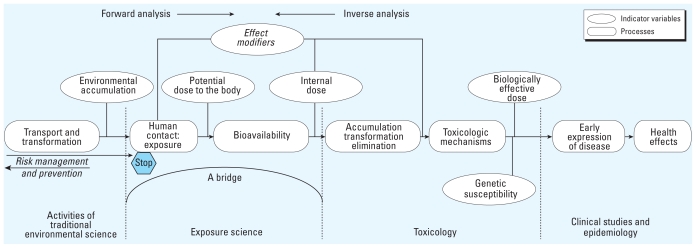
Process continuum from contaminant emissions to a health effect and application to risk reduction and prevention strategies (adapted from Lioy 1990, 1999).

Exposures measured for these six purposes capture Wayne Ott’s fundamental exposure variable: contact that occurs with a toxicant in any setting that a person encounters throughout the day (Ott 1995). Contact is an essential component of the source-to-dose continuum and must be a design issue for both mechanistic and field studies in exposure science. Observational studies must establish contact frequency, duration, and intensity of exposure agents of concern. The statement by Ott is very important, and I can easily explain it for the field by augmenting the famous phrase coined by Paracelsus—“The dose makes the poison”—to read, “The exposure provides the dose that makes the poison.” This concept is essential for improving efficiency of risk management of toxicants and for fully establishing the place of exposure science within the disciplines associated with environmental/occupational sciences (Figure 1).

### Exposure and other modeling

Exposure models have evolved from models used in the environmental sciences and have been extended to dose estimation by employing pharmacokinetic processes. They have been designed to define population exposures as well as individual personal exposures (Arnold and Price 2007; Duan 1991; Georgopoulos and Lioy 2006; Graham and McCurdy 2004; Jayjock et al. 2007; McKone 1991; Ott et al. 2007; U.S. EPA 2009). Thus, exposure models and systems use models from other fields, such as those that estimate the movement of pollutants through the environment, and human activity and physiology (Anderson and Woessner 1992; Zannetti 1990).

**Figure 2:**
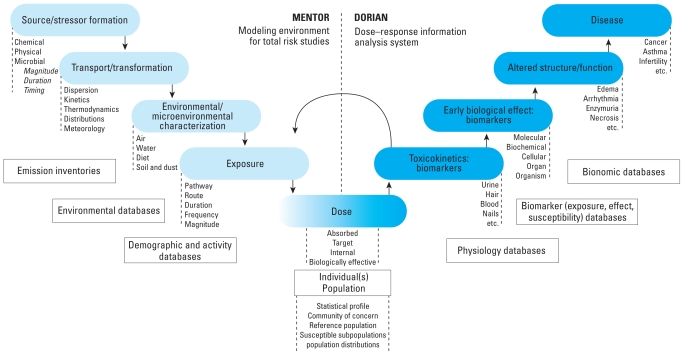
A source-to-dose modeling system: MENTOR/DORIAN (adapted from Georgopoulos 2008).

Forward-direction prognostic models developed in exposure science can be used to either generate hypotheses or test the validity or the utility of observational studies to represent exposures to larger populations or to evaluate the validity of the measurement results. A schematic representation of the entire system of variables from source to disease is found in Figure 2. This approach can be simplified to address specific problems. The general process can focus exploratory studies to examine various relationships and design new laboratory or field studies to generate as well as test hypotheses on exposure and exposure–response relationships. However, resources need to be provided to develop such tools and to engage toxicologists in the selection of the most important acute long-term effects in order to help improve risk assessment.

Results from individual observational exposure studies are often used to generalize results to larger populations using statistical and distributional modeling of exposures. Examples include the U.S. EPA’s Stochastic Human Exposure and Dose Simulation (SHEDS) and LifeLine (Burke et al. 2001; LifeLine Group 2007; Price et al. 2003; Zartarian et al. 2006, 2008), which complement the prognostic models mentioned above on source-to-dose relationships (Georgopoulos and Lioy 2006).

Current modeling systems in exposure science can characterize processes shown in Figure 1, because many environmental models can simulate the processes and impacts of pollutants. For example, the impact of the sources of a toxicant could be estimated by air or water emissions models. The transport/transformation and accumulation parts of the continuum explain both physical and chemical processes by using models frequently employed in engineering; included are fate and transport models that estimate the contaminant concentration at a location for a point in time. Again, such models do not provide estimates of exposure. The exposures are estimated using an exposure model; then, by including pharmacokinetic processes, one can estimate the time-based distribution of the levels (dose) of the toxicant and its metabolites in the body. Many types of exposure models in use are described by Georgopoulos and Lioy (2006). The use of such systems for exposure reconstruction was mentioned above, and future needs are found in Georgopoulos et al. (2009). However, the development of innovative models that reduce uncertainties in exposure characterizations can help reduce uncertainties in the design of future observational studies.

## The Future: Examples of Directions and Uses of Exposure Science

Two questions that frequently arise about exposure in the fields of environmental health sciences and risk management are “What does one do with exposure information?” and “What role does exposure science play in regulation and prevention beyond analyses of observational data and their interpretation?”

### What does one do with such information?

Observational exposure information has been used to support risk assessment, epidemiology, and exposure mitigation. Although each is an important activity, none are routinely used to prevent the toxicant from becoming a problem in the first place. The reason is that although toxicologic studies are completed on the materials used to produce a product, exposure studies are usually implemented after the toxicant has been used in commerce, when contact with the contaminant (e.g., asbestos, fuel additives, lead-laden paints) is occurring among segments of the population. For example, in risk assessments, the analyses completed in an exposure assessment are used to characterize the risk of disease within a population. Usually these exposures are employed to calculate a daily dose. The values are matched with information on the hazard of a toxic agent to establish a potential risk. Of course, this should occur before the toxicants are present in environmental media and exposure pathways are established. However, this process seems to occur only in a perfunctory manner by considering only the most obvious pathway, not necessarily at the most important time: before a product is introduced into commerce (e.g., nanoparticles). The above process should include a comprehensive exposure characterization before materials are introduced into consumer products.

Currently, in the absence of mechanistic or observational exposure data, environmental-quality data (e.g., air and water) are used as surrogates for exposure for established health standards. Thus, most of the health standards are defined in terms of the environmental media concentrations and are indirectly related to exposure. The latter is typically accomplished by estimating or evaluating the high-end (or theoretically most exposed) individual. With the collection of data on actual exposures and using them in predictive exposure models, the results can reduce uncertainties and exposure misclassification. One such example is the use of environmental-quality data in the preliminary evaluation of hazardous waste sites (U.S. EPA 1991). These may be fine for a preliminary evaluation; however, after the initial or preliminary screening assessment, exposure characterizations are essential but not routinely employed during the selection of remediation options or postremediation. In many cases, postremediation sampling is conducted but does not necessarily provide data directly related to postremediation exposure (Lioy and Burke 2010).

A project that included postremediation exposure evaluation was completed by the Environmental and Occupational Health Sciences Institute (EOHSI) and the State of New Jersey around chromium sites located in residential areas of Hudson County, New Jersey. The history of the problem is interesting. From 1905 through 1975, Hudson County was a center for chromate production and manufacturing, including chrome-plated bumpers. Facilities generated > 2 million tons of waste until about 1960. Later this waste was used as apparent clean fill at residential, commercial, and industrial locations. In the late 1980s, yellowish hexavalent chromium (Cr^+6^, a carcinogen) crystals appeared on walls of homes, schools, and other buildings, and there were yellow/green blooms of hexavalent chromium outdoors. A photo was published by Pellerin and Booker (2000). The dust was sampled in many homes that were built on or next to land contaminated with chromium waste. (Lioy et al. 1992). A variety of exposure pathways and levels of chromium in urine were examined, and it was found that people living in homes adjacent to or on a site had higher indoor levels and urine levels of total chromium. (Methods were not sensitive for Cr^+6^.) The high levels were attributable to resuspended particles, dust blown off the surface of the waste sites with high chromium, or chromium tracked indoors by residents, friends, and pets. In the end, the approximately 38 residential chromium sites were remediated to the known standard.

Uncharacteristic of most hazardous waste cleanups, about a year later the State of New Jersey funded the return of EOHSI to these homes to complete postremediation dust sampling. The results showed that the levels of chromium in house dust decreased to background levels after this period of time. Thus, the measurements successfully tested the hypothesis that the primary inhalation and ingestion pathways for residential exposure had been removed from these neighborhoods after remediation (Freeman et al. 1995, 1997). This is an example of the need to characterize pre-and postremediation exposures and should be considered when the United States reauthorizes the Superfund program or when other countries reevaluate their programs. It is not sufficient to complete long-term groundwater monitoring alone; it is equally important to show the community that there are no high exposures in the neighborhood after remediation. In addition, it is a quantitative measure of accountability that can demonstrate the success of a remediation and is easily understood.

Drinking water standards in the United States and Europe are the closest to exposure standards, because they are related primarily to the ingestion route of exposure. However, human exposure data identified other routes of significant exposure to particular pollutants found in drinking water. For example, drinking water standards did not take into account originally all uses of water in the home including showering, bathing, and food preparation. The showering issue was studied directly by Jo et al. (1990), who actually measured the exposure to chloroform that had accumulated in shower water as a residual from the chlorination process. To the surprise of many, the dose from dermal and inhalation exposure during one 10-min shower was equivalent to the dose received from drinking 2 L of water from the same tapwater source per day. Thus, those who showered longer and more frequently each day would have higher exposures. This led to reconsideration of tap water as the sole basis for drinking water standards. It was no longer reasonable to say only “Do not drink the water.” In many cases, for example, with benzene contamination, one should just not *use* the water. This point is a cornerstone for the innovative use of exposure science to implement prevention strategies.

The National Ambient Air Quality Standards or guidelines in the United States and in other countries, respectively, are usually indirectly related to exposure, because the levels are taken at a monitoring site designed to be representative of the general location of an urban or suburban population and not meant to represent actual high or low exposures or provide near-fenceline estimates of pollutant impacts (NRC 2004). Thus, populations at highest risk can be missed while attaining compliance with a standard. However, the good news is that the use of highly uncertain exposure metrics has been effectively addressed for ambient fine particulate matter and ozone standards (Foley et al. 2003; U.S. EPA 2008b).

### Management and reduction of exposure

Observational exposure data and information have been used to support risk assessment, epidemiology, and exposure mitigation. Although each is an important activity, none are routinely used to prevent toxicants from becoming a problem in the first place. Exposure studies are usually implemented after the toxicant has been released into commerce and contact is already occurring among segments of the general population. There have been few instances where efforts and resources have been specifically redirected from a small incremental exposure to a toxicant source outdoors to a larger incremental indoor or personal source of exposure to the same agent. This was illustrated by Wallace (1989) on the significance of benzene exposure from cigarettes versus emission from automobiles. However, even today, the merits of continued benzene reduction in gasoline are still under consideration (IEc 2009). Insufficient attention is being given to continued reduction/elimination of benzene exposure from tobacco smoke.

Some observational situations and simulation studies of human exposure have prompted or re-enforced efforts to intervene and mitigate or remove the toxicants—for example, in environmental tobacco smoke, asbestos, lead in dust, World Trade Center dust, indoor pesticides, trihalomethanes, polychlorinated biphenyls, polybrominated diphenyl ethers, hexavalent chromium, and methyl *tert*-butyl ether in reformulated gasoline (Gurunathan et al. 1998; Gustafson et al. 2005; Hawley 1985; Heinrich et al. 2005; Khoury and Diamond 2003; Korrick et al. 2000; Lioy et al. 1994, 2002, 2006; Wallace 1989; Weisel et al. 1993; Xu and Weisel 2005). The unfortunate aspect of these situations and others is the fact that most can be lumped under headings of unanticipated or unintended consequences, and the subsequent actions taken were necessary to clean up a toxicant or mitigate the human health effects rather than prevent them. Further, if you examine this brief list closely, it includes materials used in personal products, fire retardants, construction and architectural materials, pesticides, production wastes, and fuel additives. Thus, one can come in contact with a broad range of toxicants during daily activities and associated behaviors, and it should be a primary focus for applications to collect exposure information before the release of a product that includes a specific toxicant into commerce. In some cases, the product would still be necessary, but adequate education of the public about exposure needs to be provided. It is not sufficient to just list a common toxicant as a hazard.

As mentioned above, biomonitoring has increased in occupational and environment settings (ACGIH 2009; Barr et al. 2005; NRC 2006). For exposure science, these measurements provide a direct link between exposure and the dynamic processes of contact and the kinetic processes that define the levels or the form of a substance after it has been metabolized and redistributed, bioaccumulated, or removed from the body. The use of biomonitoring in exposure science is important, because the results can identify highly exposed or unexposed individuals when compared with a population distribution. The CDC has taken the step of developing distributions of measured values for a suite of metals, organics, and other species in the blood and urine of individuals within the National Health and Nutrition Examination Survey (CDC 2009). These data can be used to determine whether population members have relatively high or low concentrations of a toxicant at the time when the samples were taken and analyzed. The results, however, must be evaluated in the context of the specific exposure and biologic sample. For example, a high value of a pollutant does not necessarily mean that a person is at high risk if the pollutant is rapidly cleared from the body (i.e., in minutes to hours). In these cases, the elevated level could reflect a single exposure resulting from refueling an automobile or stripping furniture, rather than the average level of an ongoing exposure. On the other hand, a biomarker could reflect long-term bioaccumulation of a toxicant that could result in adverse health effects. Thus, the relevance of a single biomarker measure also needs to be considered in relation to the type and severity of a potential disease end point.

Biomonitoring is frequently included as part of observational studies of exposure. Examples include NHEXAS, TEAM, pesticide studies on farms and in schools and residences, and the National Children’s Study (NCS), to identify a few (Barr et al. 2005; Burse et al. 2000; Hoffmann et al. 2000a, 2000b; Landrigan et al. 2006; Needham et al. 2005; Sexton et al. 1995; Wallace et al. 1986). However, without other information, the results are not easy to interpret for the study participants, and they cannot lead directly to the implementation of an intervention strategy. As a result, one concern about biomonitoring is its utility in epidemiology and risk assessment beyond stating that one has been exposed and that the levels may be associated with a health outcome.

Ideally, biomonitoring requires the collection of other exposure information to identify the routes and the pathways of exposure and to eliminate sources. This complex problem has led to research programs that are beginning to develop theoretical reconstructive modeling tools to retrace the biomonitoring results backward to the routes of exposure. Inversion algorithms are required that include pharmacokinetic processes associated with the time course of accumulation of a toxicant. The goal is to eventually use many of these biomarkers as public health standards, which are coupled with other exposure information to achieve successful interventions (Dixon et al. 2009; Freeman et al. 2001a, 2001b; Rhoads et al. 1999; Weis et al. 2005). The example most frequently referred to is blood lead (CDC 1991; Dixon et al. 2009; Freeman et al. 1997; NRC 1993). A current and future challenge will be how the combination of biomonitoring, in-home measurements, and extant environmental data [as assembled and integrated in a exposure index (EI)] can be linked effectively to define exposures in the NCS (Lioy et al. 2009).

Another type of observational study that can be used to improve risk characterization and reduction is the simulated observational exposure study. Simulated exposure studies use scripted technician activities in locations representative of potentially high-exposure situations. Thus, the exposures simulate reality, but they are being measured by a scripted worker and not a volunteer subject. Other examples would be estimates of dietary intake of toxicants measured in foods bought at the grocery store and toxicants found in a local water supply. Simulation tools were employed in the 1980s to examine dermal retention of pesticides using a fluorescent tracer (Fenske et al. 1986a, 1986b) and later to simulate automobile exposure by tracking measurements in a car cabin during repeated drives along a stretch of highway (Lawryk and Weisel 1996, Stieb et al. 2008).

For inhalation exposures, the release of inert tracer gases, for example, perfluorocarbon tracers in an area of concern for exposure, such as a busy street canyon (Lioy et al. 2007), is an effective tool. On release of a tracer, technicians use scripted activities to represent potential patterns of exposure over a defined period of time, including before, during, and after a release. The results are analyzed for location and intensity of contact over time. The article (Lioy et al. 2007) discusses use of the technique in homeland security and disaster response situations. It can be used to estimate the potential magnitude of exposure to people in harm’s way and the potential hot-zone areas for emergency responders and the general public. It is a technique that still needs development, as discussed by Rodes et al. (2008), because it can also be employed to examine near-roadway exposures. It is important to make sure that the scripted techniques are selected after careful consideration of the population whose exposure is to be evaluated in a study.

### What role does exposure science play in situations beyond observational analyses and their interpretation?

The answer to this question encompasses new directions for exposure science research and enhances the contributions of exposure science to risk management and prevention programs in all countries. More important, the answer will also move the basic science components of the field to include anticipatory or intervention research and engineering and will require a heavy emphasis on source-to-dose modeling (Lioy 1998). This new direction will focus on minimizing the potential impact of unanticipated or unintended consequences or poorly designed products or applications on the general public or the general population.

The fundamental science issue is to understand contact, but the ultimate goal is to prevent exposures by examining the potential for contact and exposure before large-scale contacts among members of the general public occur. The approach includes some aspects of the precautionary principle, because the results will be used to prevent severe or irreversible harm to the public. This does not mean, however, that studies will deliberately expose individuals to products or the constituents of products before they are introduced to the market. Such studies are unethical and just poor science. The goal is proactive in nature such that the research would be designed to achieve the scientific consensus needed to show that significant harm would not occur using the product made with the specific chemical or other material before it is placed in commerce. This would be in contrast to the current practice of looking at emissions after the materials are in commerce, such as in recent studies by Gewurtz et al. (2009) that examined perfluoroalkyl contaminant emissions during carpet installation and by Imm et al. (2009) that examined the release of PBDEs from products currently used in homes.

A proactive exposure science research agenda to improve product certification by minimizing releasable toxicants would prevent contact before it can occur. Such research would involve completing simulations of potential emissions during the routine use of the product when new or as it ages, including scenarios for actual product use and not just within the manufacturer’s suggested guidelines. The results could couple source-to-exposure models with dose models to determine the potential impact on humans from acute or long-term exposures. The latter would employ the knowledge obtained from observational studies on activities and behavior to simulate contacts with new materials or products prior to introduction into commerce. The efforts would focus on prevention, not postproduction mitigation.

In some ways, the seeds have already been planted to implement this approach; however, currently there is no systematic effort to implement such a procedure by industry and governments around the globe. For example, there have been testing programs for emissions of various compounds by materials used in construction and architectural uses and home products [ANSI (American National Standards Institute) 2007; ASTM (formerly American Society for Testing and Materials) 2006; California Department of Health Services (CAL/DHS) 2004; European Committee for Standardization 2002]. Further, we understand many of the behaviors and activities that lead to exposure. However, at the present time these approaches are not used in combination within carefully designed testing programs to prevent exposures.

The following is a very simple hypothetical example of a prospective approach that uses information and tools from toxicology, exposure science, and hazard assessment. Issue: A semivolatile material, X, is being considered for use in a commercial product.

First, the following baseline toxicologic considerations are needed to frame the potential exposures:

- What is the toxicology and hazard of X compound in its raw form?
- Will it remain as compound X or be transformed to an intermediate or another compound in final form; in the transformed state, what are the added acute or long-term hazards?

The question becomes, Is the material of such a highly toxic nature that it should not even be considered for the intended use—a consideration that is currently part of the European Regulation for Registration, Evaluation, Authorization and Restriction of Chemicals (REACH) objectives (Van der Wielen 2007). This would seem to be an obvious requirement, but there are many examples of the failure to consider this in product development. Examples are lead-bearing pigments in paints applied to children’s toys, lead-laden paint in artificial turf products, outdoor residential mosquito misters, and formaldehyde in home insulation. REACH is actually developing exposure scenarios that should cover the life cycle of a toxicant, including exposure to the consumer [European Chemicals Agency (ECHA) 2008].

The next steps would be the proactive exposure science evaluation. This would be accomplished by *a*) determining the form of the final product, and how it will be used, and *b*) developing a protocol to test the product under typical use conditions (new and the aged product). Included would be the co-emission or release with other materials in the product. The plan would be to

- Conduct experiments on direct emissions of the compound X and transformed products for various uses, and their mobility.
- Couple emission results with typical activities and a variety of behaviors to estimate exposure during product use.

Depending on the material, the above activities include air emissions tests and dermal- or ingestion-related contacts. In the case of compound X, this would be air emissions or surface deposition. Further, the controlled tests for release and exposure need to be designed and conducted on both new and used products for the behaviors and activities of interest.

Final steps in the process would be to *a*) apply the results to exposure-to-dose models, similar to the Modeling Environment for Total Risk (MENTOR) approach (Figure 2) (Georgopoulos 2008), but simplified and standardized for product evaluations, and *b*) define the potential contact and exposure to compound X or final form.

The source-to-dose modeling should extend the results to typical and extreme conditions of use to examine the potential for acute exposure and long-term exposure. The estimated exposures need to be translated into estimated doses and used in conjunction with hazard assessments to determine the potential for excessive acute or long-term risks.

This simple example can have many potential permutations and a range in approaches for use with individual products. This approach to exposure science is clearly proactive and for the express purpose of prevention. Further, it links exposure science directly to the outcomes of toxicologic studies and hazard assessments. Over the years, too many compounds with great intrinsic value have been removed because of occupational or environmental exposure. That situation probably could have been avoided had the applications been limited to very specific and high-value applications designed to minimize the risk for acute or chronic health outcomes. The good news is that the technology is available or can be developed to minimize risk in specific situations, and the chemical industry has become much better at determining which chemicals are highly toxic. The problem is the lack of a framework that is acceptable to government agencies and translatable across industrial sectors that can be systematically applied before products are delivered to the marketplace. A current product problem is Chinese wallboard used in residential settings (Congressional Record 2009).

Finally, the above example would not eliminate the need for observational studies; however, they could be employed more judiciously to resolve legacies and conditions associated with current products on the market and prevent the inevitable unintended consequence of accidental or deliberate exposures.

## Conclusions

The field of exposure science has many tools to observe and characterize exposure for epidemiologic studies and risk assessments. Further, the field provides fundamental information needed to understand the dynamics and kinetics of contact and the resulting occupational or environmental exposures. The approaches have improved significantly since the early 1980s and continue to build on a scientific foundation based on theoretical and laboratory studies. The need to link exposure science to risk management and intervention is well known, but additional resources are required to ensure that the field plays a more pivotal role in preventing or mitigating exposures. This will also lead to better simulation protocols and models to characterize contact under many types and varieties of conditions before they occur as well as increase the use of exposure science regulatory strategies that prevent contact across populations stratified by age, sex, susceptibility, and culture, thus stimulating innovation. Focused and more enriched observational studies can be designed to evaluate models and improve the understanding of the levels of contact and the intensity of exposure in populations, for example, the NCS. Eventually, the general uses of exposure research will be appreciated as more than as a support tool for epidemiology or risk assessment. However, resources specifically directed toward exposure science–related research and training is needed to make this potential a reality.
